# Hyaluronic acid as an adjunct to microfracture in the treatment of osteochondral lesions of the talus: a systematic review of randomized controlled trials

**DOI:** 10.1186/s12891-022-05236-6

**Published:** 2022-04-02

**Authors:** Julian E. Dilley, Joshua S. Everhart, Robert G. Klitzman

**Affiliations:** Department of Orthopaedic Surgery, Indiana University School of Medicine, Indianapolis, Indiana USA

**Keywords:** Osteochondral lesions of the talus, Hyaluronic acid, Microfracture, Injection

## Abstract

**Background:**

Osteochondral lesions of the talus (OLT) are common after ankle trauma. Studies have shown that bioactive substances, such as hyaluronic acid (HA), alone, or in combination, with surgical treatment could improve cartilage regeneration and repair, but the effect of HA on patient reported outcomes is unclear.

**Methods:**

Literature searches were performed across four databases (PubMed, SPORTDiscus, Scopus, and The Cochrane Library) for randomized controlled trials in which at least one treatment arm involved use of HA as an adjunct to microfracture to treat patients with OLT. Primary outcomes included the American Orthopaedic Foot and Ankle Society scores (AOFAS), and the Visual Analog Scale (VAS) for pain. The level of evidence and methodological quality were evaluated using the Modified Coleman Methodology Score (MCMS).

**Results:**

Three randomized studies were eligible for review with a total of 132 patients (35, 40, 57 patients, respectively) and follow-up ranged from 10.5 to 25 months. Utilization of HA at the time of microfracture resulted in greater improvement in AOFAS scores compared to microfracture alone. The pooled effect size was moderate (Standardized Mean Difference [SMD] 0.45, 95% Confidence Interval [CI] 0.06, 0.84; *P* = .02) and between-study heterogeneity was low (I-squared = 0%). Utilization of HA during microfracture also led to greater improvement in VAS-pain scores compared to microfracture alone. The pooled effect size was very large (SMD -3.86, 95% CI -4.75, − 2.97; *P* < .001) and heterogeneity was moderate (I-squared = 69%).

**Conclusion:**

Hyaluronic acid injection as an adjunct to arthroscopic MF in OLT provides clinically important improvements in function and pain at short-term follow-up compared to MF alone. Future longer-term follow-up studies are warranted to investigate the durability of MF with HA for treatment of OLT.

## Background

Osteochondral lesions of the talus (OLT), defined as lesions of the cartilage layer and underlying subchondral bone, are one of the major challenges in orthopedic surgery [1]. These lesions can occur in up to 70% of ankle injuries [1–3] and are common among athletes [4]. Lesions are treated non-operatively or operatively. Non-operative treatment is appropriate in smaller lesions [5, 6]. In those with osteochondral fragments acting as loose bodies in the tibiotalar joint, and in those that fail nonoperative treatment, surgical management is indicated. Various surgical management strategies have been described and include excision, debridement, microfracture (MF), autologous osteochondral implantation, particulate juvenile cartilage, and autologous chondrocyte implantation [4, 6]. Of the various options, MF is often first-line given its limited invasiveness and relatively low postoperative morbidity [7, 8]. MF has shown patients have an excellent outcome in up to 72% of cases [9–12]. However, there is the concern of poor-quality fibrocartilage regeneration after MF in patients who are overweight or have extensive cartilage damage at the time of injury [7], which may be why early post-operative outcomes tend to deteriorate at later follow-up periods [9–12].

Recently, there has been growing interest in utilizing biologic compounds in addition to surgery to improve clinical outcomes in patients undergoing surgery for OLT and other cartilage conditions such as osteoarthritis. One such compound is hyaluronic acid (HA), which is produced by fibroblasts, synovial cells, and chondrocytes and is present as a major component of synovial fluid and cartilage. Recent evidence in an equine model showed that synovial fluid levels of HA were depleted after OLT [13]. In a recent systematic review, intra-articular HA has shown promise in alleviating symptoms of osteoarthritis of the ankle when compared to rehabilitation and sham injections [14], and has been shown to lead to improvements in ankle function scores [15]. However, there have been mixed results regarding symptomatic relief with HA compared to intra-articular saline injections. One small randomized controlled trial showed improvement in pain and ankle function [16], but this was not seen in a larger randomized trial of similar design [17]. For knee osteoarthritis, intra-articular HA has had mixed results ranging from ineffective [18], to being highly effective at moderate to long term follow-up [19–21] [22–25] for symptomatic management, and has been associated with decreased markers of cartilage degradation [26]. This may be due to its ability to suppress interleukin-1β mediated expression of matrix metalloproteinases under inflammatory conditions [27, 28], reduce reactive oxygen species generation by synovium [29, 30], reduce chondrocyte apoptosis, and dampen inflammatory cytokines in a molecular weight dependent manner [30, 31]. In osteochondral lesions of the knee, animal models have shown that HA alone, or in conjunction, with various scaffolds could increase the rate and amount of hyaline-like cartilage formation, and decrease chondrocyte apoptosis and improve glycosaminoglycan content [32–35]. In addition, a rabbit model of osteochondral lesions treated by MF, addition of HA hyaline-like cartilage, decreased osteophyte, and synovial inflammation [36].

There has been a paucity of high-quality comparative studies investigating the effects of HA at the time of microfracture in the treatment of OLT. Recent case series have shown generally favorable improvements in pain and ankle function scores at short to midterm follow-up in patients with OLTs undergoing HA treatment [37, 38]. In addition, non-randomized cohort studies have revealed improvement in pain and increased ankle function in OLT lesions treated with microfracture and HA at short to mid-term follow-up [39, 40]. However, there has been a lack of high quality randomized controlled trials comparing the usage of HA as an adjuvant to microfracture in OLT until recently [41–43]. The purpose of this study is to systematically review the best available randomized comparative research to determine the effect of HA plus MF versus MF alone on patient-reported pain and function for treatment of OLT.

## Methods

This systematic review was written following the guidelines for Preferred Reporting Items for Systematic Reviews and Meta-analysis (PRISMA) [44].

### Eligibility criteria

Articles were selected for inclusion with the following inclusion criteria: clinical studies that assessed the effect of HA on patients with talar osteochondral lesions undergoing microfracture, and other comparable treatments were allowed if HA combined with microfracture was one of the study groups. Additional requirements were studies designed as randomized controlled trials (RCTs), prospective cohort studies, retrospective cohort studies, studies that included a control or comparison group, and studies conducted in groups > 16 years of age. Articles were restricted to those written in English. Exclusion criteria were as follows: reviews, case reports, case series, studies with a lack of clinical outcomes, and non-clinical studies.

### Search strategy

A literature search was conducted in four databases (PubMed, SPORTDiscus, Scopus, and The Cochrane Library) for clinical studies that used microfracture and HA to treat OLT. Search terms input into each search engine were: (Osteochondral lesions OR OLT) AND (Talus OR Talar) AND (hyaluronic acid OR HA OR hyaluronate). Study abstracts were first screened. Articles that passed screening underwent full-text analysis to determine if they met eligibility criteria.

### Study outcomes of interest

Relevant data pooled from each study article were as follows: patient age, patient sex, study design, and the outcome measures American Orthopedic Foot and Ankle Society (AOFAS) Ankle/Hindfoot Scale (AHFS), and the Visual Analog Scale (VAS) for pain. The AOFAS/AHFS scores are a validated scoring system of a patient’s function of the ankle and hindfoot [45, 46]. It is scored on a 100-point scale with a higher score indicating higher function. This system considers a score of ≥90 points as excellent, 80–89 as good, 70–79 as fair, and ≤ 69 as poor. A ten-point VAS score was used to quantify patient-assessed pain, in which a score of 0 represents no pain, and a score of 10 points represents maximum pain.

### Appraisal of evidence

Quality of the included studies was assessed based on their level of evidence (LOE) using criteria published by the Journal of Bone and Joint Surgery [47], and methodological quality of evidence (MQOE) using the two-part Modified Coleman Methodology Score (MCMS) [48]. The first part of MCMS, Part A, assesses the study characteristics, and the second,part B, appraises the outcome criteria and the subject selection process (Table 1). Studies are scored 0–100 with an MCMS of < 55 considered poor, 55–69 fair, 70–84 good, and 85–100 excellent.

**Table 1:** Group demographics and study characteristics

Author, Year	No. of Ankles	Intervention	Comparator(s)	Average Age ± SD	Average Follow-Up	LOE	MQOE
Gormeli et al., 2015 [41]	40	MF/HA group Arthroscopic MF and subsequent HA injection	MF/PRP group Arthroscopic MF and subsequent PRP injection MF/saline group Arthroscopic MF and subsequent saline injection	MF/HA group 39.7 ± 8.7 years MF/PRP group 38.6 ± 9.1 years MF/saline group 40.3 ± 9.4 years	15.3 months (range, 11–25)	1	81
Doral et al., 2012 [43]	57	MF/HA group Arthroscopic MF and subsequent weekly HA injection for three weeks	MF group Arthroscopic MF alone	MF/HA group Not reported MF group Not reported Combined 40.5 ± 13.0 years	≥24 months	2	83
Shang et al., 2016 [42]	35	MF/HA group Arthroscopic MF and subsequent weekly HA injection for three weeks	MF group Arthroscopic MF alone	MF/HA group 34.7 ± 8.7 years MF group 36.6 ± 10.7 years	MF/HA group 10.4 months (SD, 1.3) MF group 10.7 months (SD, 1.1)	1	77

*Abbreviations*: *SD* Standard deviation, *LOE* Level of evidence, *MQOE* Methodological quality of evidence, *MF* Microfracture surgery, *HA* Hyaluronic acid, *PRP* Plate-rich plasma

### Data analysis and statistical methods

Baseline scores for AOFAS/Ankle and Hindfoot Scale and VAS pain scores were pooled and compared between studies. The AOFAS total score values utilized in the meta-analysis were imputed from separately reported AOFAS pain and functional sub-scores (these two sub-scores together composed the AOFAS total score) by Doral et al. [43] The pooling of AOFAS and VAS pain scores was conducted for at baseline in each study and compared at final follow-up between studies. Statistical analysis was performed using a standard software package (STATA 15.1, Statacorp, College Station, TX). A random effects meta-analysis was performed using the DerSimonian and Laird method [49]. A random effects meta-analysis accounts for between-study heterogeneity and approximates a fixed effects meta- analysis when heterogeneity is low. Treatment effect size was reported as the Standardized Mean Difference (SMD) (also referred to as Cohen’s d) [50]. A small effect is defined by Cohen as SMD = 0.2, medium effect as SMD = 0.5, and large effect as SMD = 0.8 [50]. Between-study heterogeneity was reported as Higgins I-squared, with a lower I-squared value indicating less heterogeneity [51]. Significance was set at *P* < .05.

## Results

### Search results

**Fig. 1:**
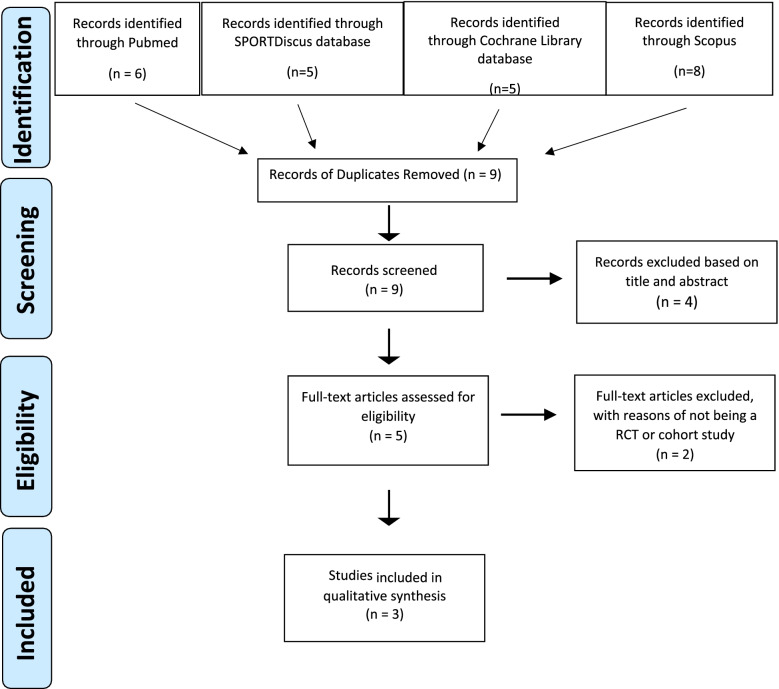
PRISMA (Preferred Reporting Items for Systematic Reviews and Meta-Analyses) flowchart of studies

After screening for inclusion and exclusion criteria, three clinical studies were included for systematic review [41–43]. A PRISMA flow diagram summarized the literature search results (Fig. 1).

### Study characteristics

Of the three articles meeting inclusion criteria, two of them met the level I evidence criteria [41, 42], and the third one met the level II evidence criteria (Table 1) [43]. Regarding the quality measurement of the trials by MQOE, all three studies were of good quality (81, 83, and 77, respectively) (Table 1). A total of 132 patients underwent arthroscopic microfracture with or without adjunct HA injections (Table 2). The average age of the participants was 38.9 (± 7.8) years; there were 61 females (46.2%) and 71 males (53.8%) in the cohorts; the medial talus was involved in 88 (71.54%) cases, and the lateral talus was involved in 35 (28.4%) cases; and the size of the lesions were less than 2 cm on average across all studies [41–43]. Baseline characteristics regarding age, sex, and lesion location were not different between the randomized treatment groups in two studies [41, 42], but these characteristics between randomized groups were not reported in one study [43]. The studies also restricted immediate postoperative weight-bearing. Time to full weight-bearing differed between the studies with patients returning to full weight-bearing in the third week after surgery in Doral and colleagues’ study, in the eighth week in Shang and colleagues’ study, and between fourth and sixth week in Gormeli and colleagues’ study [41–43]. In the HA treatment arms, a single HA injection was administered 24–36 h after surgery by Gormeli et al. [41], whereas a series of three weekly post-operative HA injections were performed by Shang et al. [42] and Doral et al. [43] The control group for Gormeli et al. received a sham saline injection, whereas the control groups for Shang et al. [42] and Doral et al. [43] had MF surgery alone without any injection.

**Table 2:** AOFAS/Ankle hindfoot scale scores

Author, Year	Study Group	No. of Ankles	Average Preoperative Score ± SD	Average Postoperative Score ± SD	*P* Value	
					Preoperative vs. Postoperative	MF/HA vs. MF
Gormeli et al.,2015 [41]	MF/HA MF/PRP MF	14 13 13	44.9 ± 9.2 43.6 ± 7.6 42.7 ± 7.1	75.1 ± 9.5 85.1 ± 6.1 68.3 ± 10.1	<.005 <.005 <.005	<.005
Doral et al.,2012 [43]	MF/HA MF	41 16	38.8 ± 9.1^a^ 44.1 ± 7.3^a^	61.9 ± 9.1^a^ 59.8 ± 9.3^a^	<.001 <.001	>.05
Shang et al.,2016 [42]	MF/HA MF	17 16	66.7 ± 4.1 65.2 ± 4.7	87.6 ± 7.6 80.8 ± 8.5	<.001 <.001	>.05

*Abbreviations*: *AOFAS* The American Orthopedic Foot and Ankle Score, *SD* Standard deviation, *MF* Microfracture surgery, *HA* Hyaluronic acid, *PRP* Plate-rich plasma

^a^Imputed values for AOFAS total score. Values were reported separately as AOFAS pain and functional subscores by Doral et al.

### Quality of studies

All studies included in this review were considered as a high LOE. In addition to LOE, MCMS can be used to evaluate the methodological quality of included studies to allow for a more nuanced evaluation [48]. The MQOE of the three studies were rated good by this methodology.

All the studies had differing weights for the final MQOE score per part A and part B. Regarding part A, the studies achieved low to moderate ratings for sample size at follow-up (35–57 subjects), and high ratings for patient compliance (100%), confirmation of diagnosis by radiograph and/or MRI, and adequate descriptions of surgical techniques and postoperative rehabilitation. The follow-up period was short in all the studies (up to 2 years) [41–43]. Regarding part B, full scores were achieved for outcome criteria and subject selection subheadings. Outcome measures were well defined and assessed by validated measures (AOFAS and VAS), with high recruitment rates. Multiple studies have reported high reliability and sensitivity of VAS for pain and AOFAS/ankle and hindfoot scale for functional measurements [45, 46, 52]. Gormeli and colleagues reported observer blinding for outcome assessments, but Shang and Doral did not [41–43].

The high LOE and “good” MQOE ratings of the three studies in this review further supported the reliability of evidence regarding the use of HA as an adjunct to MF for OLT.

### Functional outcomes

**Fig. 2:**
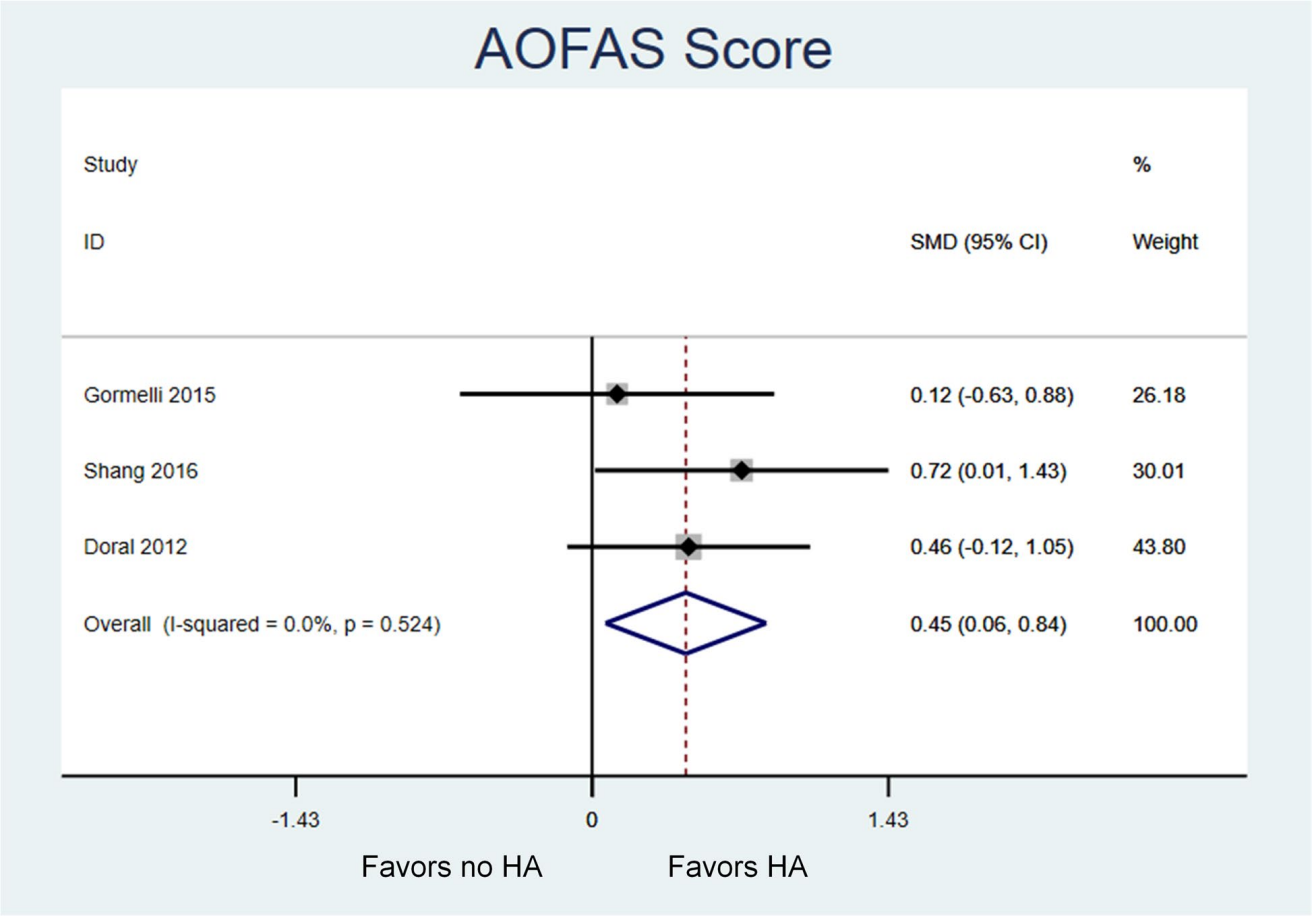
Meta-analysis of the effect of microfracture of talar OCD lesions on AOFAS scores, with versus without the addition of HA. Higher AOFAAS scores represent better function. There is a moderate size effect in favor of utilizing HA (SMD 0.45, 95% confidence interval 0.06, 0.84; *P* = .02) with low heterogeneity between studies (I-squared = 0%)

Table 2 displays baseline and follow-up values for functional assessments using the AOFAS Ankle and Hindfoot Scale from each study. Patients in all groups had significant improvement in AOFAS scores from baseline to final follow-up [41–43]. In the pooled analysis, there was a significant and moderate sized effect in favor of performing microfracture with HA rather than microfracture alone (Standardized Mean Difference [SMD] 0.45, 95% Confidence Interval [CI] 0.06, 0.84; *P* = .02) (Fig. 2). The effect was observed consistently across the studies (Higgins I-squared = 0%).

### Pain outcomes

**Fig. 3:**
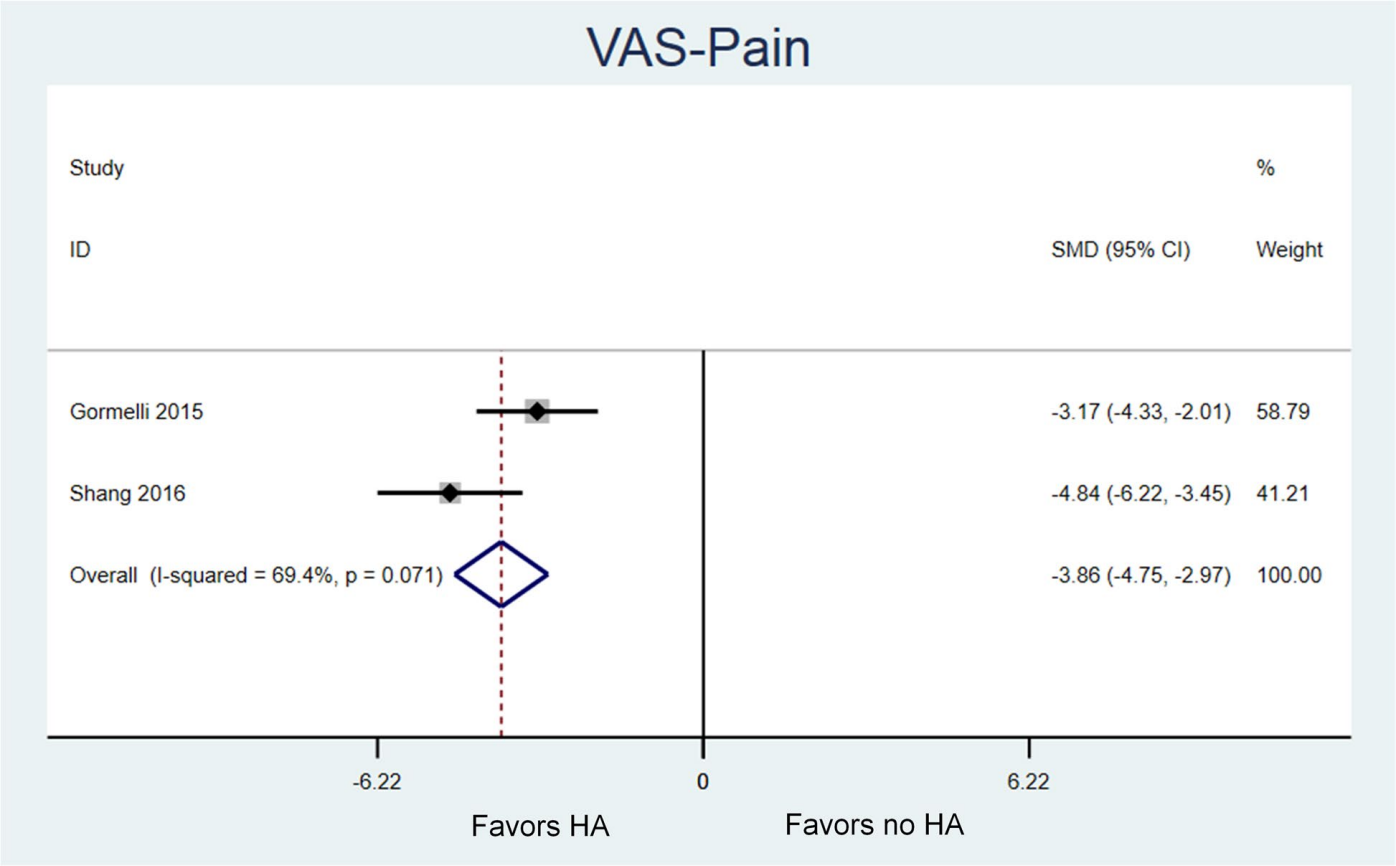
Meta-analysis of the effect of microfracture of talar OCD lesions on VAS-pain scores, with versus without the addition of HA. Lower VAS-pain scores represent less pain. There is a very large effect in favor of utilizing HA (SMD − 3.86, 95% confidence interval − 4.75, − 2.97; *P* < .001) with moderate heterogeneity between studies (I-squared = 69%)

Baseline and follow-up values for VAS scores were summarized in Table 3. Baseline VAS-pain scores were only available for analysis from two studies [41, 42]. Patients in all groups had significant improvement in VAS-pain from baseline to final follow-up [41, 42]. In the pooled analysis, there was a significant and large sized effect in favor of performing microfracture with HA rather than microfracture alone (SMD -3.86, 95% CI -4.75, − 2.97; *P* < .001) (Fig. 3). Both studies report a significantly larger improvement in VAS-pain with HA versus no HA, but magnitude of the improvement differed between studies and resulted in moderate heterogeneity in the pooled analysis (I-squared = 69%). Gormeli et al. reported an effect size favoring HA of − 3.17 (95% CI -4.33, − 2.01) [41], whereas Shang et al. revealed an effect size favoring HA of − 4.84 (95% CI -6.22, − 3.45) [42].

**Table 3:** Visual analogue scores (VAS) for pain

Author, Year	Study Group	Average Preoperative Score ± SD	Average Postoperative Score ± SD	*P* Value
				Preoperative vs. Postoperative
Gormeli et al., 2015 [41]	MF/HA MF/PRP MF	7.8 ± 0.9 8.0 ± 0.7 7.7 ± 0.7	3.3 ± 1.0 2.4 ± 0.9 4.5 ± 0.9	<.005 <.005 <.005
Shang et al., 2016 [42]	MF/HA MF	6.1 ± 0.7 6.2 ± 0.8	2.1 ± 1.3 3.1 ± 1.6	<.001 <.001

*Abbreviations*: *SD* Standard deviation, *MF* Microfracture surgery, *HA* Hyaluronic acid, *PRP* Plate-rich plasma

### Complications

Complications of HA injections include risks of joint swelling and post-injection pain. They appeared to be positively correlated with molecular weight of the HA injected in a recent meta-analysis [14]. Doral and colleagues reported no early or late complications in any patient [43]. Shang and colleagues also reported no complications including post-operative stiffness, deep vein thrombosis, infection, and neurovascular injury [42]. Gormeli et al. listed no complications in their study cohorts [41].

## Discussion

The findings of the current systematic review with meta-analysis show that HA in addition to MF for treatment of OLT lesions results in improved pain and function at short-term follow-up compared to MF alone. The magnitude of improvement in pain scores particularly with the addition of HA to MF is clinically important. In other studies, HA has been shown as a promising treatment modality in osteoarthritis, and osteochondral lesions of the knee by reducing VAS and increasing function in human studies [14, 38, 53–55]. Also, there has been evidence in animal models of higher quality hyaline cartilage being generated after osteochondral lesions when scaffolds were combined with HA [32–34], which may lead to a longer-term relief of pain and potential decrease for progression of arthritis when compared to fibrocartilage generated from traditional microfracture alone, especially in obese patients [7].

There are many hyaluronic acid compounds on the market today with variations in molecular weight, viscoelasticity, and other rheological properties. These variations in HA products may lead to differences in biologic activity. Huang et al. demonstrated that effects of IL-β and TNFα were suppressed by HA in a molecular weight dependent manner with higher molecular weight preparations leading to a larger decrease in levels of inflammatory molecules and downstream MMPs [56]. However, the effect of molecular weight on clinical outcomes, especially in osteoarthritis has been conflicting [56, 57]. Due to this variability in outcomes, it is important to discuss the preparations utilized in the included studies. Two of the studies included in this review utilized higher molecular weight preparations of HA [41–43]. Gormeli et al. administered 2 ml of Synvisc®, Shang et al. administered 2.5 ml of ARTZ® [42, 43]. However, Doral et al. did not specify the type of HA used in their study. Patients in that study did receive a half dose of HA that had a concentration of 25 mg per 2.5 ml [41]. As such, it is difficult to assess if each study had similar compounds injected intraarticularly, which may affect the ability to evaluate the efficacy of HA as an adjunct to microfracture.

Recommended improvements for future studies in this area would be the blinding of observers, and assessing patients at later follow-up periods. Longer-term follow-up is particularly important to assess the durability of HA and MF as basic science evidence has shown the potential for high-quality hyaline cartilage generation by MF coupled with HA versus the lower quality generated fibrocartilage by MF alone [32–34]. Additional examinations to assess the quality of repair tissue and regeneration of hyaline cartilage would provide more information regarding the actions of HA, alone or in combination with other biological treatments, in cartilage regeneration.

### Limitations

Several modalities were utilized to mitigate bias in this systematic review. The PRISMA methodology for systematic reviews was conducted to reduce review bias [44]. Inclusion criteria, exclusion criteria, and outcome measures of interest of data extraction were predetermined to reduce literature and data extraction biases. Despite the small sample sizes of the included studies, the treatment effect of HA was large enough to detect clinically important improvements in pain and function with low to moderate heterogeneity among the included RCTs. However, two of the included studies did not include saline injection as a sham control, which might introduce a potential bias toward larger observed effect sizes in favor of HA injection in these studies due to a lack of a placebo effect of a sham injection. The results of the current review provide no insight into the long-term efficacy of HA as an adjunct to MF for OLT. It is unclear whether HA has any treatment benefit in addition to MF at three or more years follow-up.

## Conclusion

Hyaluronic acid injection as an adjunct to arthroscopic MF in OLT provides clinically important improvements in function and pain at short-term follow-up compared to MF alone. Future longer-term follow-up studies are warranted to investigate the durability of MF with HA for treatment for OLT.

## Data Availability

The datasets used and/or analyzed during the current study are available from the corresponding author on reasonable request.

## Abbreviations

OLT: Osteochondral lesion of talus
MF: Microfracture
HA: Hyaluronic acid
RCT: Randomized Controlled Trial
PRISMA: Preferred Reporting Items for Systematic Reviews and Meta-analysis
AOFAS: American Orthopedic Foot and Ankle Society
AHFS: Ankle/Hindfoot Scale
VAS: Visual Analog Scale for pain
LOE: Level of Evidence
MQOE: Methodological Quality of Evidence
MCMS: Modified Coleman Methodology Score
PRP: Platelet Rich Plasma
